# Understanding the challenges to caring for low birthweight babies in rural southern Malawi: a qualitative study exploring caregiver and health worker perceptions and experiences

**DOI:** 10.1136/bmjgh-2017-000301

**Published:** 2017-08-22

**Authors:** Marianne Koenraads, John Phuka, Kenneth Maleta, Sally Theobald, Melissa Gladstone

**Affiliations:** 1 Department of Women and Children’s Health, Institute of Translational Medicine, University of Liverpool, Liverpool, UK; 2 Department of Community Health, University of Malawi, Zomba, Malawi; 3 Department of Public Health, School of Public Health and Family Medicine, University of Malawi, Zomba, Malawi; 4 Department of International Public Health, Liverpool School of Tropical Medicine, Liverpool, UK

**Keywords:** child health, public health, qualitative study

## Abstract

**Background:**

Low birthweight (LBW) babies account for >80% of neonatal mortality in sub-Saharan Africa and South Asia and those who survive the neonatal period are still at risk of detrimental outcomes. LBW is a major public health problem in Malawi and strongly contributes to the country’s high neonatal mortality rate. We aimed to get a better understanding of the care of LBW babies in rural Malawi in order to inform action to improve their outcomes.

**Methods:**

Qualitative methods were used to identify challenges faced by caregivers and health workers within communities and at the rural facility level. We conducted 33 in-depth interviews (18 with caregivers; 15 with health workers) and 4 focus group discussions with caregivers. Interviews were recorded, transcribed and translated. Thematic analysis was used to index the data into themes and develop a robust analytical framework.

**Results:**

Caregivers referred to LBW babies as weak, with poor health, stunted growth, developmental problems and lack of intelligence. Poor nutrition of the mother and illnesses during pregnancy were perceived to be important causes of LBW. Discrimination and stigma were described as a major challenge faced by carers of LBW babies. Problems related to feeding and the high burden of care were seen as another major challenge. Health workers described a lack of resources in health facilities, lack of adherence to counselling provided to carers and difficulties with continuity of care and follow-up in the community.

**Conclusion:**

This study highlights that care of LBW babies in rural Malawi is compromised both at community and rural facility level with poverty and existing community perceptions constituting the main challenges. To make progress in reducing neonatal mortality and promoting better outcomes, we must develop integrated community-based care packages, improve care at facility level and strengthen the links between them.

**Key questions**

**What is already known about this topic?**

- In the last decade, great progress has been made in the reduction of under-five mortality; however, the reduction of neonatal mortality has been much slower and neonatal deaths are now responsible for 45% of the global under-five deaths.
- Twenty million babies are born with low birth weight (LBW) every year, the majority in low/middle-income countries. These babies account for >80% of neonatal mortality and surviving LBW babies are at higher risk of poor developmental outcomes, stunting and non-communicable diseases later in life.
- In order to reduce neonatal mortality rates and improve long-term outcomes, we need to target the care practices for LBW and premature babies and new interventions to improve their care need to be developed.

**What does this study add?**

- We explored caregivers' and health workers' perceptions and experiences around the care for LBW babies in a rural Malawian community in order to get a better understanding of the existing barriers to care and the challenges they face.
- Caregivers in a rural Malawian community have negative perceptions about LBW babies and associate these babies with being weak, malnourished, ill and developmentally delayed. The causes of LBW are thought to be related to poor nutrition and illness of the mother during pregnancy
- In a rural Malawian context, the care of LBW babies is compromised by interplay of community perceptions leading to stigma and discrimination and poverty and poor resources at facility level as well as a lack of linkage between the two systems.

**How this might impact clinical practice?**

- Integrated community-based care packages are needed that specifically target LBW babies and their families and programmes need to be developed to support these families and raise awareness in the community to help prevent stigmatisation.
- More efforts are needed to improve care for LBW babies at facility level and better linkage to the community needs to be developed for discharge and follow-up.

## Introduction

Globally, 20 million babies are born with low birth weight (LBW) each year, the majority of them in low/middle-income countries.^1^ LBW is defined as a weight at birth of less than 2500 g^2^ and can be caused by preterm delivery (ie, before 37 weeks gestation), intrauterine growth restriction or by a combination of both.^3^ LBW babies account for >80% of neonatal mortality in sub-Saharan Africa and South Asia and small babies who survive the neonatal period are still at risk of detrimental outcomes, including stunting in childhood, neurocognitive impairment and non-communicable diseases later in life.^4 5^

Although great progress has been made over the last decade in the reduction of under-five mortality, the reduction of neonatal mortality has been much slower and neonatal deaths now account for 45% of the global under-five deaths.^5–7^ In order to reduce neonatal mortality rates and improve long-term outcomes, it is crucial to target the care practices for LBW babies. There is good evidence that interventions in the postnatal period, such as Kangaroo Mother Care (KMC), immediate and exclusive breast feeding, hygienic cord care and early detection and treatment of suspected infections can improve early mortality outcomes.^8–10^ Furthermore, there is convincing evidence that adequate stimulation and responsiveness of parents in the neonatal and infant period in combination with improved nutrition can benefit early child development.^11 12^ Despite this, both funding and action are still lacking and leadership for newborn health has been poor.^13^

Malawi has a neonatal mortality rate of 22/1000 live births and preterm birth rates are among the highest in the world.^14^ Previous qualitative studies in Malawi have highlighted that poor community care practices for preterm babies are associated with a lack of knowledge on appropriate care and poverty.^15^ Since 2002, Malawi has scaled-up KMC in health facilities, and although there is a positive attitude towards the practice, compliance and continuation of KMC are hindered by a lack of support, culture and stigma.^16^ Strengthened linkages are needed between health facilities and the community to support early discharge and follow-up for these vulnerable newborns.

This qualitative study aimed to explore the perceptions and attitudes of carers and health workers towards the care of LBW babies both in the community and at rural facility level in order to understand the challenges faced in terms of providing the best care possible in and between these two settings. This will provide a better understanding of how intervention packages can be adapted in a more productive and understandable way for communities in rural African settings.

## Methods

This study used a qualitative approach in order to generate an in-depth understanding of the participants' perspectives, attitudes and experiences within their social context.^17^

The study was located in rural villages within Nankumba Traditional Authority and in Mangochi District Hospital, southern Malawi. Mangochi district has the lowest educational attainment of the country with 35.3% of women being uneducated and only 51.5% being literate.^18^ The total fertility rate in Mangochi district is 7, with a median age at first birth of 18–19 years, the neonatal mortality rate is 36/1000 live births and 9.4% of babies are born with LBW.^18^ Access to healthcare is poor with 66.5% of women reporting the distance to a health facility to be problematic. Despite this, 69.3% of women are now delivering in a health facility.^18^ The Nankumba Health Centre has a catchment population of 25 233 spread over 18 villages, and 5000 households with 1262 children less than 1 year old and 4290 children less than 5 years old (numbers from local health centre records). Mangochi District Hospital is the largest hospital in the district.

Data collection took place from May to July 2013 and April to June 2014. We used individual in-depth interviews (IDIs) in combination with focus group discussions (FGDs) to add breadth to the data and triangulate the findings.^19^ Participants were purposively selected. We introduced the study and the aims to local village chiefs, who identified a key informant, a local health surveillance assistant (HSA). The key informant identified and approached potential caregivers face to face to inform them about the study and give written information. If participants were willing to participate, the research team visited them in their home and obtained written consent before conducting the IDIs/FGDs. Health workers involved in the care for LBW babies at Nankumba Health Centre and in Mangochi District Hospital were approached and informed by a member of the research team and consent was taken before conducting the interviews. The sample size was determined by the saturation principle, data analysis was a continuous and iterative process and we stopped data collection when no more new themes emerged from the data. See table 1 for the sample characteristics.

**Table 1:** Participants involved in in-depth interviews (IDIs) and focus group discussions (FGDs)

Method of data collection	Participant characteristics	Number
IDIs		33
	Mothers of LBW babies <2 years old	18
	Health workers	15
	Clinical officers	2
	Medical assistants	4
	Nurse–midwife technicians	3
	Health surveillance assistants	5
	Hospital attendant	1
FGDs		36 (four groups)
	Mothers of LBW babies <2 years old	17 (two groups)
	Mothers of children <2 years old	9 (one group)
	Fathers of children <2 years old	10 (one group)

LBW, low birthweight.

Interviews and FGDs were conducted by a local research assistant in the local language, Chichewa. Topic guides were developed to guide interviews and were translated into the local language and then pilot tested and adjusted where necessary. Topics covered in the guide for caregivers were: perceptions of small babies, feeding and caring of a small baby, challenges and community support. The topic guide for health workers covered what happens after delivery of a LBW baby, initial care given, discharge to the community, follow-up and challenges. The interviewer was free to change the sequence of topics and follow up any answers given by the participant in order to probe for more information, explanation or description.

All interviews and FGDs took place in participants' homes or other private and confidential areas and were, after permission, recorded on a digital voice recorder. Recordings were transcribed verbatim and subsequently translated into English.

Data were analysed using thematic analysis. After thorough familiarisation with the transcripts, key themes were identified inductively and a conceptual framework was developed. NVivo qualitative data analysis software (QSR International, V.10, 2012) was used as a data management tool to assist in indexing the data. Subsequently, data were coded and further categorised. We enhanced the reliability and validity of this study by using the technique of ‘thick description’, which aims to provide a detailed description of the context and research setting.^20^ Furthermore, we used different research techniques (IDIs and FGDs) and explored the perspectives of different participant groups (male and female caregivers and health workers) to ensure comprehensiveness and triangulate our findings.^21^ The building of trust and rapport of the research assistant with the participants and parallel observations of the primary researcher further enhanced the credibility of the data.^22^

The first author, a paediatric doctor with interest and experience in international child health, was the primary researcher who conducted the field work and identified the initial themes after reading and rereading the translated transcripts. Themes were adjusted and refined after discussion with other members of the research team.

## Results

### Community understanding of LBW

To develop a contextual understanding of the care for LBW babies in a rural Malawian community, we first explored existing perceptions and beliefs of mothers of LBW babies as well as the views from other caregivers in the community and local community health workers. We explored participants' views on three different aspects: recognition, causes and long-term effects of LBW (table 2).

**Table 2:** Summary of caregivers' and health workers' existing perceptions and beliefs of LBW babies in a rural Malawian community

Recognition	Low weight; not heavy to carry; has a weight less than 2500 g
	Baby appears very small, thin and malnourished
	Baby seems unhappy
Causes	Inadequate nutrition of the mother during pregnancy
	Illness in the mother during pregnancy
	Young age of the mother
	Multiple pregnancies
	Lack of antenatal care; use of family planning
	Premature delivery
	Stress during pregnancy
	Too much physical work
	Abuses from men
Effects	Poor health and frequent illness
	Poor weight gain/stunted growth
	Developmental delay and not intelligent in school
	Early death

#### Recognition of LBW

Caregivers in the community described LBW babies subjectively. Mothers of LBW babies, as well as other mothers and fathers, reported that they recognised these babies as having very low weight and not feeling heavy when carrying them. They referred to these babies as being very small, thin, malnourished in appearance and not in good health when looking at them.

*The baby looks small and malnourished and when you carry the baby the weight is very low. [FGD, Mothers of LBW babies]*

Some mothers as well as fathers also described these babies as looking unhappy, not being happy in life and being unhappy with their parents. Community health workers used more objective measures and identified LBW babies as babies with a birth weight less than 2500 g.

#### Perceived causes of LBW

Poor nutrition of the mother during pregnancy was seen as an important factor in causing LBW. Mothers of LBW babies as well as other mothers, fathers and health workers thought LBW was due to eating inadequately during the pregnancy.

*‘Maybe when I was pregnant food was not enough, we get advice that we should eat a balanced diet, but because it was not like that, that might be the cause that I have a small baby.’ [IDI, Mother of LBW baby]*

*‘If the mother is not eating good food during pregnancy it will be difficult for the baby to be born with good health.’ [FGD, Fathers]*

*‘Most of the times it happens because when the mother was pregnant, she was not getting nutritious foods for the baby to feed on through the mother, so because of a lack of foods the baby is born with low birthweight.’ [IDI, HSA]*

A few caregivers and health workers also expressed that LBW can be related to the mother having a certain disease such as HIV.

*‘Diseases are numerous like now we have AIDS, if you have the disease the baby in the womb is affected and also during the pregnancy the mother is stressed of thinking what the baby will be like due to the disease in the mother’s body.’ [FGD, Mothers]*

Other factors reported by health workers were young age of the mother and having multiple pregnancies.

*‘Most of the time its multiparity and also, you know this is a village and most of the women get pregnant when they are not at the recommended age of 18 yet, that also contributes’. [IDI, MA]*

Several mothers also mentioned that LBW was caused by a lack of family planning, delivering early, stress and doing too much work. One father reported the lack of antenatal care as a cause.

*‘Most of the times these problems happen because the mother did not get advice, because during pregnancy she should attend antenatal clinic at an early stage.’ [FGD, Fathers].*

One mother reported that abuse from men can have a negative impact on the mothers’ eating habit, thus indirectly causing LBW.

‘*Sometimes abuses from men can cause a woman not to eat, and then in the end she will give birth to a LBW baby.’ [FGD, Mothers]*

#### Effects of LBW

There was a general perception in the community that LBW babies are weak and have poor health. Participants described these babies as getting sick very often with diseases such as malaria, cough, diarrhoea, vomiting and fever.

*‘I can tell through my experience, small children are hard to take care of, they are often sick with pneumonia, sometimes malaria with high fevers. They also vomit if they are exposed to cold.’ [IDI, Mother of LBW baby]*

*‘They get sick more often because their body defence is weak against disease. The babies’ health is nothing but misery.’ [FGD, Fathers]*

Several caregivers and health workers also expressed concerns about slow weight gain and stunted growth and long-term developmental problems such as a delay in sitting and walking, problems with hearing and problems with intelligence in school.

*‘Normal size children have fast growth where as low birthweight children are retarded in growth and malnourished.’ [IDI, Mother of LBW baby]*

*‘They are not intelligent at school even when they grow up, the thinking capacity is lower than those with the right weight.’ [FGD, Mothers of LBW babies]*

*‘There are myths and misconceptions that this child would not grow and would not be clever, and that this child would not benefit the world.’ [IDI, HSA]*

One mother also mentioned that these babies have a higher risk of dying.

*‘Sometimes the baby can die because of not following what the health workers advise us and then there will be no improvement in the body weight…so maybe she will not live long and she may die at any time.’ [IDI, Mother of LBW baby]*

### Challenges to the care of LBW babies

To gain a better understanding of the barriers to the care of LBW babies in a rural Malawian community, we explored the challenges faced by their caregivers as well as health workers who are looking after these babies.

### Challenges for caregivers

#### Discrimination/stigma

Discrimination and stigma were described as a major challenge faced by carers of LBW babies in the community. Mothers of LBW babies mentioned being accused of not taking care of themselves and their babies and being associated with having diseases such as HIV. Other mothers and fathers in the community described that these mothers of LBW babies feel ashamed and stay inside their homes and do not attend women groups and under-five clinics because of fear of being mocked and laughed at. Heath workers also confirmed that these mothers face discrimination in the community and reported that when following up a LBW baby, people in the community are often suspicious as to the cause of the baby’s difficulties and think that the baby may have a disease such as HIV.

*‘When they see us they point fingers at us and say that we don’t eat adequately.’ [FGD, Mothers of LBW babies]*

*‘…when given birth to these small babies they refer to us as being sick or infected*…’* [IDI, Mother of LBW baby]*

*‘They meet problems as they become the talk of the village and they are not free to associate themselves in women groups.’ [FGD, Mothers]*

**‘*In the community there are people who have their beliefs that when a mother has given birth to such a baby, then they just regard the baby as a dead one. So these mothers are scared that when they go to the community that people will discriminate them or they will be laughed at.’ [IDI, NMT]*

Several mothers of LBW babies and other mothers and fathers also described how even when LBW infants grow up they still face stigma and mockery in their daily lives with potential detrimental effects on their physical health and development.

*‘…they are beaten up by friends who see them to be weak.’ [FGD, Fathers]*

*‘They are segregated and teased by friends and do no play well with other kids.’ [FGD, Mothers of LBW babies]*

*‘They are given names as short person, stunted and some call the kid a witch or wizard.’ [FGD, Fathers]*

#### Feeding problems

Problems related to feeding LBW babies were described as another major challenge by both caregivers and health workers, especially the inability of these babies to take milk from the breast. The majority of mothers as well as several fathers and health workers described that these babies are too weak to suck and that they get tired easily; also, a lack of breast milk due to lack of food for the mother was reported to be a problem. They described managing these problems in a number of ways which included: forcing the baby to suck, cup feeding the baby, buying milk, giving complementary foods and using traditional medicine.

*‘When he was born the breastmilk was not coming out, I tried forcing him to suck, but his jaws were not ready.’ [IDI, Mother of LBW baby]*

*‘We squeeze the breast to produce milk and put it in a cup and give it to the baby.*’ *[FGD, Mothers of LBW babies]*

*‘There is lack of food for the mother, we don’t provide formula milk so the milk depends only on the mother, so if the mother is not eating well the breastmilk will be insufficient and it will be hard for the baby to grow.’ [IDI, MA]*

*‘When the mother sees that the breastmilk is not coming out then she starts complementing the baby with other foods.’ [IDI, HSA]*

#### High burden of care

The care of a LBW baby was seen as an extra high burden on families where day-to-day life issues around survival are already high. LBW babies were perceived to be more difficult to look after as they need extra care, have difficulties feeding and are often sick. Several mothers reported that they were always busy with the care of the baby and therefore had to leave their other household chores and work. Health workers also reported that caring for a LBW baby is a burden and there is very little support for caregivers/mothers, making it difficult to provide their babies with the optimal care.

*‘Taking care of a small baby is a problem. For instance I did not have time to do other work except concentrating on exclusive breastfeeding*…*though with scarcity of food in the house from morning to evening this life seems tough.’ [IDI, Mother of LBW baby]*

*‘They don’t get enough care when at home. Maybe the mother is just alone at home and there is no one to help her in caring for the baby. Then it is hard for the mother to be working while carrying the baby, so in the end they expose the baby, instead of putting the baby on the stomach, like the kangaroo. So if the baby is cold, then it does not grow and dies in the end before his/her time.’ [IDI, MA]*

### Challenges for health workers

#### Lack of adherence to counselling

Health workers both at facility level and in the community described how they counselled mothers of LBW babies on best practices in caring for their babies. This included advice on exclusive and frequent breast feeding, keeping the baby warm and KMC, but also nutrition for the mother and identification of illness in the baby. Health workers at facility level complained about the lack of adherence to the advice they provided which they saw as inevitably leading to negative outcomes for babies.

*‘We can advise the mothers, but for them to follow on those advises…’ [IDI, HSA]*

*‘The challenge that we face like in the wards, is that most of the mothers don’t care for these babies. For instance you can tell them to be covering the babies, but you will find that they don’t have things for covering the baby. So when some of them are discharged the care continues to be poor at home and at the end, we lose the baby as in the baby dies.’ [IDI, CO]*

Health workers explained that mothers’ poor understanding and low education levels, poverty, a lack of support and traditional beliefs in the community are related to the poor adherence to their counselling.

*‘Most of the women have not gone far with their school, so because of their education they don’t understand what we are telling them.’ [IDI, NMT]*

*‘The challenge is that most mothers do not understand what we say whilst here in the hospital, so some mothers run away secretly without us knowing, and sometimes the baby dies on the way.’ [IDI, MA]*

*‘…as I have said earlier on we do advise them, but once they go in the community they start listening to their fellow mothers on what to do with their babies and they forget what the doctors advised them…’ [IDI, HSA]*

Analysis of responses from both IDIs and FGDs with caregivers showed that mothers are actually well informed about the international recommendations on exclusive breast feeding in the first 6 months of life, the need for LBW babies to feed frequently and the importance of keeping these babies warm and clean. However, both caregivers and health workers explained that limited resources and lack of support meant that carers were not always able to follow the advice provided.

#### Lack of resources

Health workers also complained how limited resources challenged the quality of care for LBW babies. They reported that it was difficult to keep these babies warm with the limited facilities in the community and due to a lack of electricity and incubators at the health facilities.

*‘As a health centre we don’t have a lot of supplies, for example resuscitation materials we don’t have them and we don’t have incubators, because we don’t have electricity.’ [IDI, MA]*

*‘We don’t have enough resources that can help us in managing those LBW babies. We try our best with what we have, but we are supposed to have maybe clothes that we can give to them or a separate room for kangaroo*…*’ [IDI, MA]*

Health workers also reported a lack of space and equipment for the large numbers of babies and highlighted that low staffing levels were really problematic both in community and health facilities.

*‘Sometimes we don’t have the equipment to check the babies’ temperature and sometimes during the weekends, because there were few workers, the babies were not weighed and sometimes those who were supposed to be given drugs did not receive them because of insufficient drugs.’ [IDI, NMT]*

#### Discharge procedures, follow-up and community links

Health workers at rural facility level explained that LBW babies were discharged from the health centre when they reached a certain target weight; however, the weight described varied from 1800 to 2500 g according to different health workers. Furthermore, a great heterogeneity in follow-up practices was described; including follow-up in the hospital by HSAs in the community, who then care for this baby until it reaches a normal weight and follow-up in antenatal clinic at 6 weeks, when the baby goes for his/her first vaccinations. Some mentioned that there is no follow-up for these babies at all and that they

*‘just tell them to come back for a check-up if there are other problems like if the baby is sick or if the baby is not breathing well’. [IDI, MA]*

*‘The time to discharge the mother depends on how the baby’s weight is increasing. Usually when its 1.9 we are supposed to discharge the mother with education which she is supposed to continue when she goes home. For those whose weight is 2–2.4 kg we just teach them to be doing kangaroo at home but we tell them that they have to come back to the hospital for review every two weeks.’ [IDI, NMT]*

*‘When the baby has been discharged from the hospital the baby goes to the community, where the HSA for that community is alerted that they have a low birthweight baby in the community who is supposed to be cared of until the baby reaches a normal weight.' [IDI, HSA]*

## Discussion

**Figure 1:**
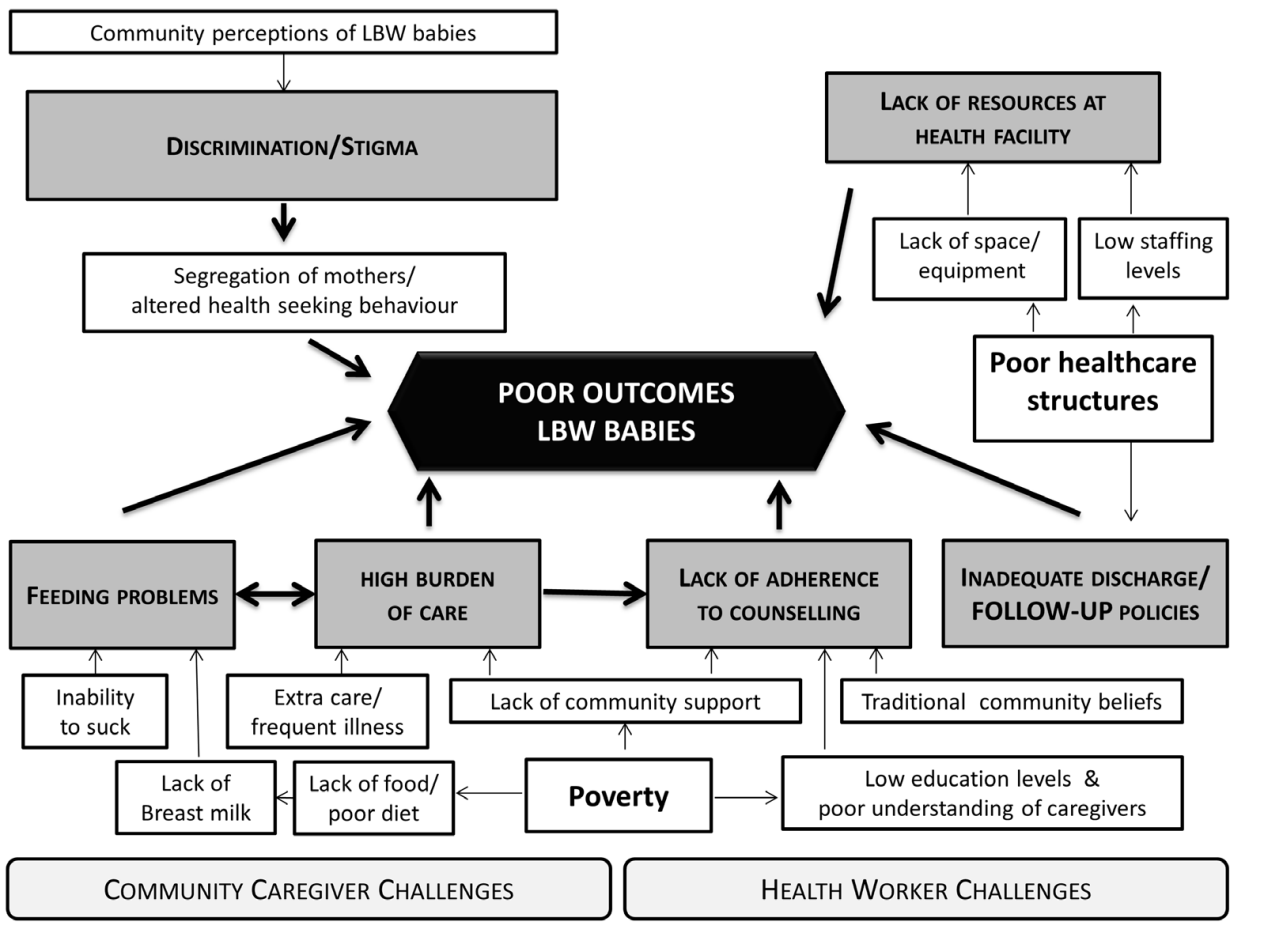
Pathways between caregiver and health worker challenges leading to poor outcomes for low birthweight (LBW) babies in rural southern Malawi.

In this study, we explored perceptions and experiences around the care for LBW babies in rural communities and health facilities in southern Malawi. Our findings illustrate how in a rural Malawian context poverty and existing perceptions interplay at the community level to shape experiences and reaction to LBW babies and are compounded by limited resources at facility level, see figure 1.

Discrimination and stigma relating to having a LBW baby has emerged as a central theme in our work and poses a major challenge to mothers with LBW babies. Our study has illustrated how this may lead to segregation of these mothers and that this may have potential detrimental effects on the babies’ health. This is similarly described in qualitative work looking at experiences of KMC in Malawi.^16^ Negative community perceptions as to the causality of having a LBW baby seem to lead to this discrimination and stigma. These perceptions include a generally negative perception of these babies as being malnourished and unhappy. In our study, caregivers also associated LBW with being weak and ill and with poor longer term outcomes such as not being intelligent and suffering mockery by peers. The perceived causes of LBW reported in our study (poor maternal nutrition, illness in pregnancy, poor antenatal care, early labour, having multiple births, young maternal age, stress, heavy physical work and abuse) are in line with results of previous studies in similar settings^23^; however, other studies also mentioned the will of God, supernatural powers and witchcraft as causes.^15 24–26^ Most of the causes reported in ours and other studies were maternal and imply that community members blame the mother for the baby being small.

Our study has also highlighted the major challenge for caregivers in feeding and caring for LBW babies. Mothers struggle to exclusively breast feed due to poor sucking of the baby, a lack of breast milk and the high burden of other household chores. We know that exclusive breast feeding has a protective effect on overall mortality and morbidity from pneumonia and gastrointestinal infection, so it is important that we target these specific issues much more for mothers in these settings.^27–32^

Despite health workers reporting their counselling of mothers on supporting their LBW baby (breast feeding, keeping the babies warm and recognition of illness), they also blamed mothers for lack of adherence to their advice. This is likely to be shaped in part by poor education and traditional beliefs, but our findings show challenges faced by caregivers are compounded by a general lack of support for mothers who have high expectations placed on them from health workers and communities. This was also described in a study on preterm birth in Malawi which reported the high burden of care leading to neglecting business, farming and household chores.^15^ Furthermore, health workers complained about a lack of resources to deliver appropriate care of these babies. In the community, fundamental resources such as electricity are lacking and at facility level there are issues with staffing, space and supply of medicine.

In summary, our study highlights the multiple challenges to the care of LBW babies in the community, many which stem from the perception and understanding of the causality of having a LBW baby. These findings are likely to have an impact on caregivers’ motivation and home care practices and our results confirm the importance of taking these local beliefs into account when developing new interventions and public health campaigns that target neonatal survival. Furthermore, we evaluated the care pathway for these babies, starting in the rural health facility and identified several challenges for health workers at facility level such as a lack of resources and problems with counselling. We also identified that there is no clear structure for discharge and referral of these vulnerable LBW babies and there is no structured follow-up at the health facility or in the community. It is clear that poverty and poor healthcare structures underlie most of the challenges.

The Newhints trial in Ghana has shown promising results of a home visit intervention to increase the uptake of KMC in a rural community setting^33^ and Save the Children previously piloted a programme in Malawi that used mobile phones to facilitate the follow-up of LBW babies.^34^ These are some encouraging examples of interventions that have a potential to improve the care for LBW babies in rural African settings.

As this was a qualitative study, our purposively selected study sample is not statistically representative, and it’s therefore not possible to generalise these results to the wider population. The limitations of this study are a potential bias in the inclusion of the caregivers as they were identified by our key informant, the local HSA. The HSA has the duty of educating and following up LBW babies, so those caregivers who have not been reached by the HSA were excluded and could have had different perceptions and experiences. Also, we did not include caregivers of LBW babies who have died. Another limitation is the possible occurrence of the Hawthorne effect which refers to the fact that participants might alter their responses to the expectations of the observer.^35^

## Conclusion

It is crucial to improve the care practices for LBW babies in order to reduce neonatal mortality and improve long-term outcomes for these babies. Our results show that more action and funding are needed to develop integrated community-based care packages that target these babies and their families specifically. Furthermore, there is a need to raise awareness in the community to reduce stigmatisation of mothers with LBW infants. Care at rural facility level needs to be improved with better resources and well-educated staff and more efforts need to be made to improve the linkage between the community and health facility. Programmes should aim to improve healthcare and nutrition in pregnancy and the neonatal period, but also support good parenting and opportunities for education. This will enable these infants to develop and grow to their full potential and will help to contribute in efforts to stop the intergenerational transmission of poverty.

## References

[1] UNICEF, WHO. Low birthweight: country, regional and global estimates. New York: United Nations Children’s Fund and World Health Organisation, 2004.

[2] WHO. Intermational statistical classification of diseases and related health problems, tenth revision. Geneva: World Health Organization, 2011. 3376487

[3] Kramer MS Determinants of low birth weight: methodological assessment and meta-analysis. Bull World Health Organ 1987;65:663–737. 3322602 PMC2491072

[4] Walker SP, Wachs TD, Grantham-McGregor S, et al Inequality in early childhood: risk and protective factors for early child development. Lancet 2011;378:1325–38. 10.1016/S0140-6736(11)60555-2 21944375

[5] Lawn JE, Blencowe H, Oza S, et al Every newborn: progress, priorities, and potential beyond survival. Lancet 2014;384:189–205. 10.1016/S0140-6736(14)60496-7 24853593

[6] Liu L, Johnson HL, Cousens S, et al Global, regional, and national causes of child mortality: an updated systematic analysis for 2010 with time trends since 2000. Lancet 2012;379:2151–61. 10.1016/S0140-6736(12)60560-1 22579125

[7] Lawn JE, Blencowe H, Kinney MV, et al Evidence to inform the future for maternal and newborn health. Best Pract Res Clin Obstet Gynaecol 2016;36:169–83. 10.1016/j.bpobgyn.2016.07.004 27707540

[8] Darmstadt GL, Bhutta ZA, Cousens S, et al Evidence-based cost-effective interventions: how many newborn babies can we save? The Lancet 2005;365:977–88. 10.1016/S0140-6736(05)71088-6 15767001

[9] Lawn JE, Cousens S, Zupan J, et al 4 million neonatal deaths: When? Where? Why? The Lancet 2005;365:891–900. 10.1016/S0140-6736(05)71048-5 15752534

[10] Bhutta ZA, Das JK, Bahl R, et al Can available interventions end preventable deaths in mothers, newborn babies, and stillbirths, and at what cost? The Lancet 2014;384:347–70. 10.1016/S0140-6736(14)60792-3 24853604

[11] Engle PL, Black MM, Behrman JR, et al Strategies to avoid the loss of developmental potential in more than 200 million children in the developing world. Lancet 2007;369:229–42. 10.1016/S0140-6736(07)60112-3 17240290

[12] Engle PL, Fernald LC, Alderman H, et al Strategies for reducing inequalities and improving developmental outcomes for young children in low-income and middle-income countries. Lancet 2011;378:1339–53. 10.1016/S0140-6736(11)60889-1 21944378

[13] Darmstadt GL, Kinney MV, Chopra M, et al Who has been caring for the baby? Lancet 2014;384:174–88. 10.1016/S0140-6736(14)60458-X 24853603

[14] van den Broek N, Ntonya C, Kayira E, et al Preterm birth in rural Malawi: high incidence in ultrasound-dated population. Hum Reprod 2005;20:3235–7. 10.1093/humrep/dei208 16037105

[15] Gondwe A, Munthali AC, Ashorn P, et al Perceptions and experiences of community members on caring for preterm newborns in rural Mangochi, Malawi: a qualitative study. BMC Pregnancy Childbirth 2014;14:399 10.1186/s12884-014-0399-6 25444374 PMC4264332

[16] Chisenga JZ, Chalanda M, Ngwale M Kangaroo mother care: a review of mothers'experiences at Bwaila hospital and Zomba Central hospital (Malawi). Midwifery 2015;31:305–15. 10.1016/j.midw.2014.04.008 24908188

[17] Denzin N, Lincoln Y The discipline and practice of qualitative research Thousand Oaks CA, Handbook of qualitative research. Sage, 2000:1–28.

[18] NSO, ICF Macro. Malawi Demographic and Health Survey 2010. Zomba, Malawi, and Calverton, Maryland, USA: National Statistical Office and ICF Macro, 2011.

[19] Crabtree BF, Yanoshik MK, Miller WL, et al Selecting individual or group interviews Morgan DL, Successful focus groups: Advancing the state of the art. London: Sage Publications, 1993:137–49.

[20] Kuper A, Lingard L, Levinson W Critically appraising qualitative research. 2008 337(Journal, Electronic). 10.1136/bmj.a1035 18687726

[21] Mays N, Pope C Qualitative research in health care. assessing quality in qualitative research. BMJ 2000;320:50–2. 10.1136/bmj.320.7226.50 10617534 PMC1117321

[22] Pretty JN Participatory inquiry for sustainable agriculture. London: IIED, 1993.

[23] Imdad A, Bhutta ZA Nutritional management of the low birth weight/preterm infant in community settings: a perspective from the developing world. J Pediatr 2013;162:S107–S114. 10.1016/j.jpeds.2012.11.060 23445841

[24] Nabiwemba EL, Atuyambe L, Criel B, et al Recognition and home care of low birth weight neonates: a qualitative study of knowledge, beliefs and practices of mothers in Iganga-mayuge health and demographic surveillance site, Uganda. BMC Public Health 2014;14:1697–716. 10.1186/1471-2458-14-546 PMC4064282 24888464

[25] Tolhurst R, Theobald S, Kayira E, et al ‘I don’t want all my babies to go to the grave’: perceptions of preterm birth in Southern Malawi. Midwifery 2008;24:83–98. 10.1016/j.midw.2006.09.003 17240496

[26] Waiswa P, Nyanzi S, Namusoko-Kalungi S, et al 'I never thought that this baby would survive; I thought that it would die any time': perceptions and care for preterm babies in eastern Uganda. Trop Med Int Health 2010;15:1140–7. 10.1111/j.1365-3156.2010.02603.x 20723185

[27] Arifeen S, Black RE, Antelman G, et al Exclusive breastfeeding reduces acute respiratory infection and diarrhea deaths among infants in Dhaka slums. Pediatrics 2001;108:e67 10.1542/peds.108.4.e67 11581475

[28] Black RE, Allen LH, Bhutta ZA, et al Maternal and child undernutrition: global and regional exposures and health consequences. Lancet 2008;371:243–60. 10.1016/S0140-6736(07)61690-0 18207566

[29] Daniels MC, Adair LS Breast-feeding influences cognitive development in Filipino children. J Nutr 2005;135:2589–95. 16251616 10.1093/jn/135.11.2589

[30] Thakur SK, Roy SK, Paul K, et al Effect of nutrition education on exclusive breastfeeding for nutritional outcome of low birth weight babies. Eur J Clin Nutr 2012;66:376–81. 10.1038/ejcn.2011.182 22085870

[31] Edmond KM, Kirkwood BR, Tawiah CA, et al Impact of early infant feeding practices on mortality in low birth weight infants from rural Ghana. J Perinatol 2008;28:438–44. 10.1038/jp.2008.19 18322552

[32] Kramer MS, Kakuma R Optimal duration of exclusive breastfeeding. Cochrane Database Syst Rev 2012:CD003517 10.1002/14651858.CD003517.pub2 22895934 PMC7154583

[33] Vesel L, ten Asbroek AH, Manu A, et al Promoting skin-to-skin care for low birthweight babies: findings from the Ghana Newhints cluster-randomised trial. Trop Med Int Health 2013;18:952–61. 10.1111/tmi.12134 23731228

[34] Children S Kangaroo Mother Care in Malawi: successes, challenges and opportunities. Lilongwe, Malawi: Save the Children Federation, 2014.

[35] Holden JD Hawthorne effects and research into professional practice. J Eval Clin Pract 2001;7:65–70. 10.1046/j.1365-2753.2001.00280.x 11240840

